# Treatment-resistant schizophrenia: How far have we traveled?

**DOI:** 10.3389/fpsyt.2022.994425

**Published:** 2022-08-30

**Authors:** Ambu Pandey, Kamal Narayan Kalita

**Affiliations:** ^1^Department of Psychiatry, Maharshi Devraha Baba Autonomous State Medical College, Deoria, India; ^2^Department of Psychiatry, Lokpriya Gopinath Bordoloi Regional Institute of Mental Health, Tezpur, India

**Keywords:** treatment-resistant, treatment-refractory, neurobiology, clozapine, pharmacogenomics, precision-medicine, schizophrenia

## Abstract

Treatment-resistant schizophrenia is a lack of adequate response to antipsychotic medications resulting in incomplete functional and social recovery from the illness. Different definitions have been proposed for clinical practice and research work. Antipsychotics that are used in the management of schizophrenia mainly act on multiple dopaminergic pathways which are implicated in the development of symptoms of schizophrenia. Newer antipsychotics also are implicated to affect the serotonergic pathways. Clozapine is the only evidence-based treatment available for the management of treatment-resistant cases. Neurobiologically, there is a considerable overlap between treatment-resistant and treatment-responsive cases. The factors that are implicated in the evolution of treatment resistance are still not conclusive. These make the management of such patients a challenge. However, certain peculiarities of treatment-resistant schizophrenia have been identified which can guide us in the early identification and precise treatment of the treatment-resistant cases.

## Introduction

Schizophrenia is a serious mental disorder characterized by distortion of thought, perception of reality, difficulties in executive functions, and expression of emotions and behavior. Schizophrenia affects around 0.5–0.8% of world's population (1). Even after a century of its clinical description, schizophrenia continues to puzzle researchers with its variety of presentations and complex etiology involving the role of genetics and its interaction with environmental factors. It has always been described as a chronic illness with a relatively guarded prognosis.

## Evolution of the concept of treatment-resistant schizophrenia

Before the twenty-first century, patients suffering from psychosis or other serious mental disorder were either living on the streets or homed in asylums or jails. In the twenty-first century with the introduction of insulin shock therapy and electroconvulsive therapy, which were successful in improving symptoms of schizophrenia, there arose a possibility of remission from symptoms of schizophrenia. The discovery of chlorpromazine, the first antipsychotic medication in the 1950s, quite successfully improved the positive symptoms of schizophrenia resulting in a marked decline in long hospitalizations and the integration of people suffering from schizophrenia back to their families (2). With the development of second-generation antipsychotics, there was better control of negative symptoms in addition to the positive symptoms, with fewer side effects improving the prognosis of illness and the quality of life of patients suffering from schizophrenia.

Before the development of antipsychotic medication in the early part of the twenty-first century, the concept of treatment non-response was based on the need of placing the patient in an asylum. Cabaleiro Goas described patients with schizophrenia based on their level of social functioning. He said that complete remission in schizophrenia occurs when symptoms subside completely, or even with the defect, the affected individual can live a normal life. Incomplete remission occurred when illness or defect only allowed the patient a reduced level of social and family life and in cases with no remission, there was no improvement in symptoms and patients had to be kept in asylums (3). It is noteworthy that these earlier criteria considered the level of social and familial functioning and not only the symptomatic improvement. In the 1970s after the development of antipsychotics, quantitative criteria were included in defining treatment resistance in schizophrenia, like hospitalization for more than 2 years and persistence of positive symptoms despite appropriate treatment with antipsychotics (4).

In the 1980s, researchers began to include pharmacological treatment in their definition of treatment-resistant schizophrenia (5). Deniker et al. defined treatment resistance in schizophrenia when there was the persistence of symptoms for more than 2 years even after 6 months of treatment with antipsychotic medicines in adequate doses (6). The first scientifically acknowledged definition of treatment-resistant schizophrenia was given by Kane and his coworkers during their multicenter Clozapine Trial in 1988, where they also indicated that clozapine was the gold standard treatment for resistant cases (7). Later on, the criteria given by him underwent certain modifications with respect to the duration of treatment, number of failed trials, and pharmacological doses of drugs, which were incited by the finding that 400 mg of chlorpromazine would block 80–90% of dopaminergic receptors (8–11). This made the criteria less restrictive, and based on modified Kane's criteria, the prevalence of treatment-resistant schizophrenia reached 42.9% from 12.9% when unmodified Kane's criteria were used (12). Kane categorically separated treatment-resistant cases from non-resistance. However, this is not the case in day-to-day practice, and various levels of response are observed. He also gave much importance to positive symptoms ignoring the heterogeneity of symptoms of schizophrenia, residual symptoms, and social functioning of patients suffering from schizophrenia.

May et al. and many other researchers postulated that treatment response is not an all-or-none phenomenon, but there are different levels of responses in clinical practice (5). He also emphasized on the integration of social consequences of illness and psychosocial interventions available for the illness (13). Brenner et al. described the response to treatment in schizophrenia on a continuum ranging from clinical remission to severe treatment refractoriness where there is the persistence of psychotic symptoms with significant social and behavioral dysfunction despite continuous pharmacological and non-pharmacological interventions. They also emphasized on the fact that in some patients, the response to treatment worsens over time (2, 14). Treatment-resistant schizophrenia is defined as failure to respond to two trials of antipsychotics or adequate dose and duration other than clozapine in most clinical guidelines (15).

## Understanding treatment-resistant schizophrenia

Approximately one-third of all patients with Schizophrenia are treatment-resistant (2). Treatment resistance in schizophrenia may occur from the first episode or it may occur later in the course of the illness (16–18). The cases that are unresponsive from the beginning indicate that these cases are more severe cases with neurodevelopment underpinnings. When it occurs later in the course of illness after the initial response, it is preceded by many relapses, which can be due to non-adherence to medication or treatment discontinuation (19–22) or even co-occurrence of other illnesses including physical ones. It is important that we differentiate true treatment resistance from pseudo-resistance as early as possible so that appropriate steps can be taken to eliminate the factors leading to pseudo-resistance and improve the outcome for the patients. Pseudo-resistant cases are those who have been misdiagnosed or patients who have compliance issues or those who have received inadequate dose, duration, or attain sub-therapeutic plasma levels of antipsychotic medication, and cases with medical or psychiatric comorbidities or side effects of medications overshadowing the response to antipsychotic medications (19, 23, 24).

It is hypothesized that treatment-responsive schizophrenia is different from treatment-resistant schizophrenia (25). Also, the cases responsive to treatment at the beginning and those that are resistant from the beginning differ from each other (21). Increased dopamine activity in the striatum is the most accepted cause of the development of psychotic symptoms (26–28). Positron emission tomographic studies have shown that not all patients with schizophrenia have increased striatal dopaminergic activity. Instead, some patients with treatment-resistant schizophrenia have shown normal or even hypodopaminergic activity (25, 28, 29). Studies have also shown that treatment-resistant cases that do respond to clozapine treatment have lower dopamine synthesis in the striatum (30). This indicates that some cases of treatment-resistant schizophrenia may be neurobiologically distinct from treatment-responsive cases.

Dopamine hypersensitivity has been proposed to explain resistance, which develops in cases who were initially responsive to treatment. It proposes that dopamine receptor blockade by antipsychotic medications leads to dopamine receptor upregulation such that the patients relapse and need increasing doses of antipsychotic medication for symptomatic control. Although tolerance to antipsychotic medication doses is seen and higher doses of antipsychotic medications are required in controlling the symptoms during relapse following initial response, this theory does not completely explain the failure of response to antipsychotic medication (31–35).

Other hypothesis based on animal experiment proposes that dopaminergic hyperactivity is a secondary loss of GABA-ergic neurons, which modulates the activity of dopamine in the midbrain. Even a short duration of dopamine receptor blockade to control the effects of the hyper-dopaminergic state leads to the failure of GABA receptors to respond to its modulators, which would have otherwise corrected the fault that lead to the hyperdopaminergic state (34, 36, 37).

Dysfunction of neurotransmitters other than dopamine has also been implicated in schizophrenia. Glutamate and GABA dysregulation leading to dopaminergic hyperactivity in schizophrenia has also been proposed (38–41). Studies have shown increased levels of glutamate concentration in the anterior cingulate cortex in treatment-resistant cases but not in all cases of schizophrenia who respond to first-line antipsychotic treatment (25, 42).

Studies have also shown that genetic variants associated with treatment-resistant schizophrenia are related to dopamine-related genes. Genes involved in glutamatergic and serotonergic systems are also affected in TRS (43–45). In a Genome-Wide Association Study of Schizophrenia, it is found that the common genetic variants associated with schizophrenia were distinctively associated with treatment resistance. In previous studies, this association was not found due to a broad definition of schizophrenia that was used to conduct the studies (46).

A meta-analysis of neuropsychological functions has shown differences between treatment-resistant and treatment-responsive schizophrenia. Neuropsychological deficits in patients with schizophrenia distinguish them from healthy controls. This study even found that the treatment-resistant cases differed from treatment-responsive cases in deficits in the domain of memory and learning and also language function. In general, language and verbal intelligence deficits are not the most differentiating finding in schizophrenia compared with healthy controls; however, the deficit in this domain was the most distinguishing feature between treatment-resistant and treatment-responsive schizophrenia on neuropsychological testing (47). Neurolinguistic research may be an important area of work even in a globalized world where language and culture are continually adapting to the migration of people.

Many structural and functional differences are also seen in the brains of treatment-responsive cases of schizophrenia and non-treatment-responsive cases. Treatment-resistant cases have a greater reduction in the gray matter, especially in frontal regions, a decreased thickness of the dorsolateral prefrontal cortex, and an increased basal ganglia white matter volume in comparison to those who respond to treatment (48–51). Studies have found disrupted resting state functional connectivity in patients with schizophrenia. Functional neuroimaging techniques have found decreased global functional connectivity in treatment-resistant cases (52–54). The disruption was not only in thalamocortical circuits but also within the thalamic subregions in treatment-resistant cases (55). Although studies have found differences in treatment responsive and resistant schizophrenia, the differences are not always found which means that there is considerable overlap in the neurobiology and they are not a completely separate entity. Again it is difficult to implicate the neurodevelopmental and neurodegenerative theories in etiology specifically in treatment responsive and resistant schizophrenia.

Treatment-resistant schizophrenia affects roughly 30% of people who suffer from schizophrenia (2). In clinical practice, patients who do not respond to two adequate doses and duration of antipsychotic medication are treated as treatment-resistant cases of schizophrenia. Clozapine is the only effective medication for treatment-resistant schizophrenia (56, 57). Notably, 60–70% of cases of treatment-resistant schizophrenia respond to clozapine (58). Clozapine is, however, an underutilized medication, and nearly 50% of patients with treatment-resistant schizophrenia fail to receive a proper trial of clozapine in fear of its side effects and the need for therapeutic blood monitoring and other investigations. Precision medicine and pharmacogenomics can help to enhance the use of clozapine and also development to new medicines for treatment-resistant cases of schizophrenia (59–62). Precision medicine utilizes genetic and other biomarkers to give tailored medication to individuals, thereby increasing safety, effectiveness, and ultimately the overall outcome of health (63). Pharmacogenomics helps to understand how the entire genetic constitutions of an individual affect their response to medication whether or not the genes are involved in the pathogenesis of the disease (64). With the utilization of precision medicine, it will be possible to identify genetic variants who respond to clozapine and genes which are associated with adverse drug reactions, and it will also help in the development of new drugs. This will probably be a positive development in care of the individuals with this dreaded disabling disease in the coming years.

Clozapine is metabolized in the liver by CYP1A2 primarily. The minimum effective plasma clozapine concentration is 0.35 mg/L (65). CYP1A2 is a highly inducible enzyme making smokers, requiring higher doses of clozapine. Many variants of CYP1A2 have been identified affecting clozapine metabolism. CYP1A2^*^1F has been associated with resistant schizophrenia (66–68). CYP1A2^*^1F mutation in the region of intron 1 makes the gene highly inducible in smokers, thus quickly metabolizing clozapine and rendering them unresponsive to it (69). Single nucleotide polymorphism in the ABCD1 region of the CYP1A2 gene is also associated with response to clozapine (70). However, these studies are still in their infancy, and pharmacogenetic testing for CYP enzymes for prescribing is not recommended in current treatment guidelines.

Clozapine is an atypical antipsychotic, which acts on many neurotransmitter systems. A functional polymorphism of Ser9Gly in type 3 dopamine receptors is found to be associated with response to clozapine in many studies though it was not found to be statistically significant in a meta-analysis (71, 72). Polymorphism of serotonin receptor type 2A has been associated with response to clozapine (73). Pimavanserin is a new drug approved for levodopa-induced psychosis in patients suffering from Parkinson's disease. It has actions mainly on 5HT2A and 5HT2C receptors with no appreciable affinity for other receptors (74). Initially, it was found to be ineffective in schizophrenia when it was tried on non-treatment-resistant cases. Recently, it has been found to be effective in controlling positive symptoms in treatment-resistant non-clozapine responding cases of schizophrenia (75). The glutamatergic system has also been implicated in treatment-resistant cases of schizophrenia, where there is a normodopaminergic or hypodopaminergic state with an increased level of glutamate in the anterior cingulated cortex (25, 29). Many preclinical and human studies have found that clozapine interacts with the glutamatergic system and enhances its neurotransmission which is responsible for the clinical effects of clozapine in treatment-resistant cases. Many genes have been studied in this regard. GRIN2B gene which codes for the 2B subunit of NMDA receptor variations is being particularly studied in regard to response to clozapine (76).

Based on the neurobiological understanding of treatment-resistant schizophrenia, new drugs are being tried. Molecules such as glycine, D-serine, and D-cycloserine have been tried in schizophrenia to enhance glutamatergic neurotransmission but they were found to be ineffective. Sodium benzoate, which is a D-amino acid oxidase, metabolizes D-amino acids and activates NMDA receptors, thereby improving glutamatergic transmission, has been found to be effective in clozapine-resistant cases of schizophrenia at doses of 2 g per day in a 6-week clinical trial (77). Muscarinic receptor agonist xanomeline is a new molecule found to be successful in some studies (78, 79). Chronic inflammation and oxidative stress have also been associated with treatment resistance and response. Elevated levels of inflammatory markers have been seen in treatment-resistant cases, which can be potent targets for the utilization of immunotherapy as an adjunct in treatment-resistant cases of schizophrenia (80). To enhance the functional connectivity of neural circuits, transcranial magnetic stimulation, and transcranial direct current stimulation are being used (81, 82).

## Future perspective and conclusion

Treatment resistance in schizophrenia has manifold aspects. At present, no definition encompasses all the aspects, and this is because the current understanding of the pathogenesis of illness is not complete. The current neurobiological finding clearly indicates that treatment-resistant cases differ from treatment-responsive cases of schizophrenia. More research in the identification of clinical, genetic, imaging, and immunological biomarkers should be performed, which would help in spotting treatment-resistant cases of schizophrenia in the earliest stages and early initiation of appropriate treatment. It will also help in the application of precision medicine in schizophrenia and the quest for the discovery of newer targets and drugs for schizophrenia.

## Data availability statement

The original contributions presented in the study are included in the article/Supplementary material, further inquiries can be directed to the corresponding author.

## Ethics statement

The studies involving human participants were reviewed and approved by IEC, LGBRIMH. Written informed consent for participation was not required for this study in accordance with the national legislation and the institutional requirements.

## Author contributions

AP: first draft of the article. KK: initial communication, selected the topic and designed the article, did literature search, corrected the draft, and checked for plagiarism. All authors contributed to the article and approved the submitted version.

## Conflict of interest

The authors declare that the research was conducted in the absence of any commercial or financial relationships that could be construed as a potential conflict of interest.

## Publisher's note

All claims expressed in this article are solely those of the authors and do not necessarily represent those of their affiliated organizations, or those of the publisher, the editors and the reviewers. Any product that may be evaluated in this article, or claim that may be made by its manufacturer, is not guaranteed or endorsed by the publisher.
