# Supplementary material for: Treatment-resistant schizophrenia: How far have we traveled?
Source: Front Psychiatry. 2022 Aug 30;13:994425. doi: 10.3389/fpsyt.2022.994425 (PMC9468267; doi:10.3389/fpsyt.2022.994425)
Supplement: Supplementary file 1 [file Table_1.DOCX]

|  | Factors | Studies |
| --- | --- | --- |
| 1. | Treatment non-adherence | Emsley et al., in their study found that 97 patient of schizophrenia relapsed during their maintenance with placebo, of these, majority had been ill for many years and had multiple previous hospitalizations. 14 of these patient became non responsive to treatment during post relapse treatment. 18 patient who received paliperidone in the maintenance phase also relapsed [20].  Takeuchi et al., in their study on 130 patients of Schizophrenia or Schizoaffective disorder, who were previously diagnosed with first episode psychosis and had responded to a second generation antipsychotic but relapsed due to non-adherence, found that there was significant delay and reduction in response and requirement of higher doses of antipsychotic with which they had previously responded [21]. |
| 2. | Dopamine activity alterations | Demjaha et al., in their ^18^F-DOPA PET study that there was increased synthesis of dopamine in striatum of treatment responsive cases of Schizophrenia but not in treatment resistant group [25].  Jauhar et al., in their study on 40 patients of first episode psychosis and 14 healthy controls found an increased pre-treatment dopamine synthesis capacity using ^18^F-DOPA PET in treatment responders but not in non-responders and controls [28].  In another study by Demjaha et al., it was found that the increase in dopamine synthesis capacity in responder but not in non-responder patients of Schizophrenia and healthy controls was seen in associative and limbic regions of the striatum [29].  Kim et al., in their ^18^F DOPA- PET study found that 12 patients of schizophrenia who failed to respond to first line drug and responded with clozapine had lower dopamine synthesis capacity in striatum than in 12 patients who responded to first line drugs [30].  Chouinard et al., in 1978 proposed that hypersensitivity of dopamine receptor result from chronic dopaminergic blockade by antipsychotics result in tardive dyskinesia and drug tolerance [31].  Suzuki et al., in his study conducted on 147 patients of treatment resistant schizophrenia found that approximately 70% of patients with treatment resistant Schizophrenia have experienced one or more dopamine super-sensitivity psychosis episodes [32]. |
| 3. | Involvement of other neurotransmitter systems | Lodge et al., in their animal model of Schizophrenia found that methyazoxymethanol acetate (MAM) treated rats have reduced density of parvalbumin positive interneurons in medial prefrontal cortex and dorsal subiculum resulting in decreased functionality of prefrontal cortex and hippocampus during task performance [40].  Lodge et al., in another study found that prenatal administration of MAM resulted in adult rats with spontaneous increase in dopaminergic neuron firing in ventral tegmentum [41].  Mouchlianitis et al., in their study on 21 treatment resistant and 20 treatment responsive patients of Schizophrenia using proton magnetic resonance spectroscopy found elevate levels of glutamate in anterior cingulate cortex when compared with treatment responsive patients [42]. |
| 4. | Gene studies and pharmacogenomics | Bilic et al., in their study comprising of 173 patients of Schizophrenia out of which 92 were treatment resistant found that patients with treatment resistance had certain combination of SERT-in2 and DAT polymorphism [43]  Sagaud et al., in their study found that high frequency of catechol-o-mehyltransferase rs4680 AA genotype and rs4818 CC genotype was associated with treatment resistance in females but not in males [44]  Ruderfer et al., in their study found that treatment resistant patients of Schizophrenia had more of rare disruptive variants of antipsychotic gene targets [45]  Pardinas et al., in their genome wide association study found that common genetic variations were associated mainly with treatment resistance cases of Schizophrenia [46].  Eap et al., in their study on 4 non responding patients treated with clozapine, with low plasma levels of clozapine to have mutation in intron 1 of CYP1A2 gene (CYP1A2*1F) rendering the gene highly inducible and with ultra-rapid activity [66].  Balibey et al ., in their study on 55 patients of schizophrenia treated with clozapine found that non response to clozapine was 2.4 times higher in those patients who had CYP1A2*1F*1F genotype [67]. |
| 5. | Neuropsychological factors | In a meta-analyses of 17 studies with total 1864 participants, comparing the neuropsychological differences between treatment resistant and treatment responsive patients of Schizophrenia. Neuropsychological deficits were significant in all domains in treatment resistant cases but were most pronounced in the domain of verbal learning and memory [47]. |
| 6. | Structural and functional factors | Molina et al., in their neuroimaging study comprising of 30 treatment –resistant cases of schizophrenia, 19 non treatment resistant schizophrenia patient and 44 healthy controls found that there was significantly less gray matter volume in frontal and occipital region and more with matter volume in these regions of the brain in MRI in comparison to healthy controls which was not significant for non-treatment resistant patients [49].  Quarantelli et al., in their voxel based morphometry study comprising of 20 non responding cases of Schizophrenia, 15 responding patients and 16 healthy controls found a significantly reduced gray matter volume of bilateral superior and middle frontal gyri in non-responders [50].  Anderson et al., in their study found a significant gray matter volume reduction in frontal, temporal and occipital regions in treatment resistant and ultra-treatment resistant cases of Schizophrenia in comparison to responders and healthy controls [51].  McNabb et al, in their study using structural and resting state fMRI technique found that there was weak network connectivity in ultra-treatment resistant cases(n=16) of schizophrenia in comparison to healthy controls( n=17) [53].  Ganella et al., in their study of functional brain networks using resting state fMRI in 42 treatment resistant schizophrenia patients and 42 healthy controls found significantly weaker global brain functional connectivity [54].  Kim et al., in their study using resting state fMRI on 50 treatment – resistant patients of Schizophrenia and 61 healthy controls found an increased functional connectivity between the thalamic sub-regions and various cortical regions in case group in contrast to the controls [55] |
| 7. | Inflammatory marker studies | In FACE-SZ study by Leboyer et al, where 195 patients of Schizophrenia were recruited, 20% and 7.7% patients were treatment resistant and ultra-treatment resistant respectively and had elevated levels of interleukin (IL) -12/IL-23p40, IL-17A, iL-10, beta 2 micro-globulin, suggesting involvement of T helper 17 pathway in treatment-resistant Schizophrenia [80]. |
